# Bifid mandibular condyles: A systematic review

**DOI:** 10.4317/medoral.22681

**Published:** 2018-11-21

**Authors:** Jordi Borrás-Ferreres, Alba Sánchez-Torres, Cosme Gay-Escoda

**Affiliations:** 1 DDS. Fellow of the Master’s Degree Program in Oral Surgery and Implantology (EFHRE International University/FUCSO). Postgraduate degree on Temporomandibular Disorders and Orofacial Pain (SCOE, Barcelona, Spain); 2 DDS, MS, Master of Oral Surgery and Implantology. Associate Professor of Oral Surgery, School of Medicine and Health Sciences, University of Barcelona. Researcher at the IDIBELL Institute. Barcelona (Spain); 3 MD, DDS, MS, PhD, EBOS, OMFS. Chairman and Professor of the Oral and Maxillofacial Surgery Department, School of Medicine and Health Sciences, University of Barcelona. Director of Master’s Degree Program in Oral Surgery and Implantology (EFHRE International University/FUCSO). Coordinator/Researcher of the IDIBELL Institute. Head of Oral and Maxillofacial Surgery and Implantology Department of the Teknon Medical Centre, Barcelona (Spain)

## Abstract

**Background:**

Bifid mandibular condyle (BMC) constitutes an extremely rare disorder characterized by a duplication of the head of the mandibular condyle. Its prevalence ranges from 0.31% to 1.82% in the published literature.

**Objectives:**

The primary objective was to describe the main etiological, clinical and radiological characteristics of patients with BMCs and the existent treatment options. The secondary objective was to simultaneously include the characteristics of two new cases of BMC.

**Material and Methods:**

An electronic search in Pubmed (MEDLINE), Scopus and The Cochrane Library was carried out by two independent reviewers until April 2018. Prospective or retrospective cohort studies, case series and case reports describing clinical and/or radiological characteristics of patients with BMC were included. Registered variables were demographic, etiological factors, diagnostic exam, clinical characteristics and treatment options. The results from the articles selected were organized in a Table along with the characteristics of two new cases of BMC provided by the authors.

**Results:**

From a total of 431 articles found in the initial search, 68 articles were finally included. This systematic review included 216 patients and 270 BMC with an average age of 30.6 (SD=14.7) years and a women:men ratio of 1.4:1. Mediolateral condylar orientation was the most prevalent position (80.1%). Among cases with known etiology, 40.8% of cases had a history of traumatism, while 55.9% did not present any relevant medical background. Half of the symptomatic cases had history of trauma. The most common symptoms were hypomobility (22.7%), arthralgia (18.1%), articular noise (17.2%) and ankylosis (17.6%). Active monitoring and manufacturing an occlusal splint were the most frequent treatment options.

**Conclusions:**

BMC may have congenital or traumatic etiology. Hypomobility and arthralgia are the most frequent symptoms and treatment options are often conservative.

** Key words:**Bifid mandibular condyle, trifid condyle, tetrafid condyle, condylar orientation, ankyloses.

## Introduction

Bifid mandibular condyle (BMC) constitutes an extremely rare disorder characterized by a duplication of the head of the mandibular condyle (1-3). The actual prevalence of BMC is controversial as it widely ranges from 0.31% to 1.82% among previously published studies (4-6).

This disorder is considered to be a developmental abnormality although it has also been related to infection, trauma, condylar fractures or condylectomy (7-16). Some authors have suggested that mediolateral orientation of the condyle is associated to a non-traumatic etiology (fibrous septa), while anteroposterior position is more related to a previous trauma (17,18).

BMC can be asymptomatic or present distinct signs and symptoms such as pain, swelling, noise, hypomobility, joint block, deflection, joint luxation or even ankylosis (2,8,13,14,19-24). Asymptomatic cases often have a congenital etiology, mainly detected by a routine examination (1). On the contrary, symptomatic cases are frequently associated to traumatic events (5,19,20,25).

While a great majority of BMCs have coincidentally been diagnosed with a panoramic radiography (PAN) during a routine exam (1,12,14,19,26-31), computed tomography (CT) is considered to be the test of choice for an appropriate diagnosis (13,16,18,30,32). Accordingly, Sampaio *et al.* (33) published a retrospective study which found a 1.1% prevalence of BMC in asymptomatic patients by using CT meanwhile only half of them could be diagnosed by checking previous panoramic radiographies.

The primary objective of this systematic review was to describe the main etiological, clinical and radiological characteristics of patients with BMCs, and the existent treatment options. The secondary objective was to simultaneously include the characteristics of two new cases of BMC.

## Material and Methods

This article has been performed according to the Preferred Reporting Items for Systematic Reviews and Meta-Analyses (PRISMA) statement (34).

Prospective or retrospective cohort studies, case series and case reports describing clinical and/or radiological characteristics of patients with BMC were included. No restriction of language neither year of publication was applied. Cross-sectional studies or articles describing cadaveric samples were excluded.

An electronic search in Pubmed (MEDLINE), Scopus and The Cochrane Library was carried out by two independent reviewers until April 2018. The search strategy was (“bifid condyle” NOT “canals” NOT “third molar [MeSH]”) for Pubmed (MEDLINE) and (“bifid condyle”) for Scopus and The Cochrane Library. A manual search by reading the references of the selected studies was also performed.

The articles were initially selected by reading the title and abstract. The full text of the selected studies was then evaluated. Any discrepancies were resolved by consensus. A Cohen’s kappa was calculated to determine the interrater reliability by SPSS 22.0 (SPPS Inc. Chicago, USA). The selected articles were classified into distinct levels of evidence according to the Strength of Recommendation Taxonomy (SORT) criteria (35).

Registered variables were demographic (age, gender), etiological factors, diagnostic exam, clinical characteristics (location, condyle orientation, signs and symptoms) and treatment options. The results from the articles selected were organized in a Table along with the characteristics of two new cases of BMC provided by the authors.

## Results

**Figure 1:**
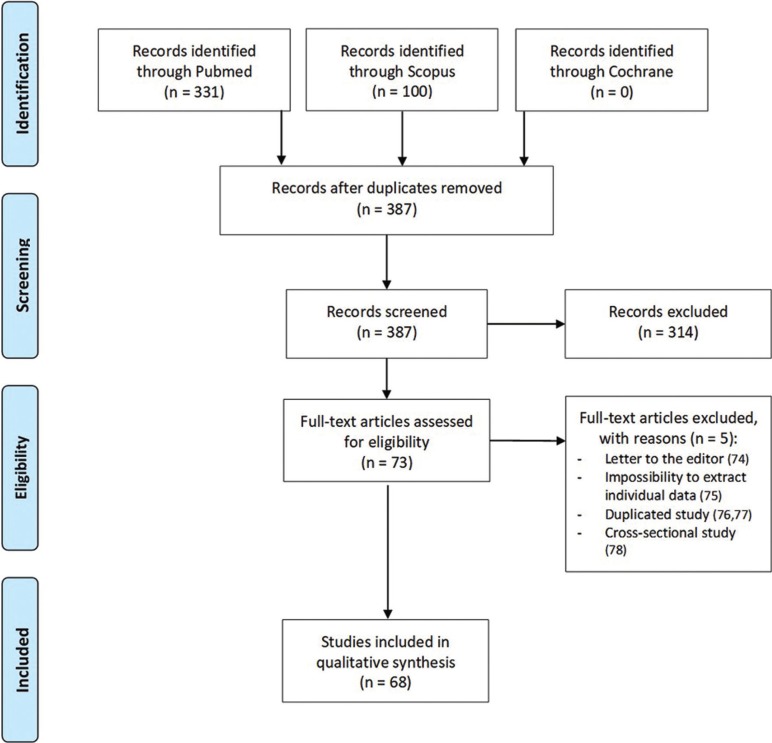
Selected articles. Flow chart of the selected articles through the systematic review process according to PRISMA guidelines.

A total of 431 articles were found in the initial search. After the elimination of duplicated and no relevant articles by reading titles and abstracts, 73 articles were full-text evaluated. Finally, 68 articles were included in the systematic review: 6 case series (4-6,5,30,36) and 62 case reports (1,2,7-14,18-29,31-33,37-73). The level of agreement between reviewers was good, with a Cohen’s kappa value of 0.7468. Figure 1 shows the flow chart of the selected articles through the systematic review process and the reasons for the exclusion of articles after the full-text evaluation (74-78). All of the selected articles had a level 3 of SORT criteria. Furthermore, 2 new case reports were included by the authors.

**Figure 2:**
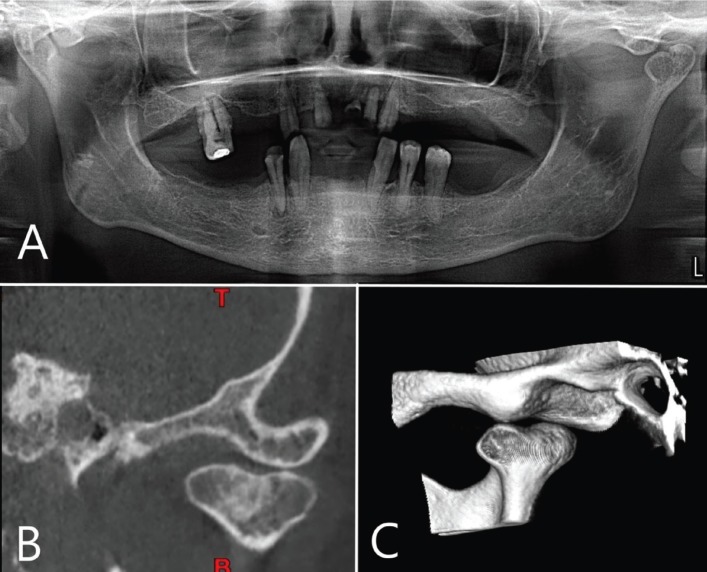
Case 1. A) Panoramic radiography shows a left BMC. B) Coronal slice from computed tomography. Mediolateral condylar position. C) 3D reconstruction. Posterolateral aspect.

**Figure 3:**
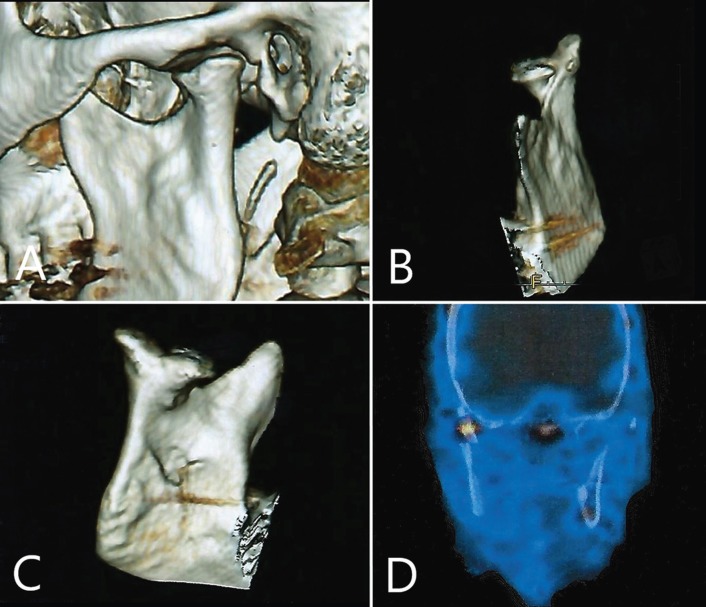
Case 2. Computed tomography 3D reconstruction. A) Sagittal view. B) Anterolateral view. C) Posteromedial view. D) SPECT frontal view.

This systematic review includes 216 patients with 270 BMC, including the two new cases provided by the authors showed in Figures 2 and 3.

 Table 1,  Table 1 continue shows demographic, clinicopathological and therapeutic characteristics of the cases included. Patients with BMC had an average age of 30.6 (SD=14.7) years old and women were more affected than men with a ratio 1.4:1. Unilateral involvement was the most prevalent although there was a remarkable proportion of bilateral BMC reaching the 25%. Mediolateral condylar position of the strictly BMC (only 2 heads) was the most prevalent among patients (80.1%). Moreover, 7 trifid and 2 tetrafid condyles were found. Among cases with known etiology, a 40.8% of cases had a history of traumatism, while 55.9% did not present any relevant medical background. Most of the included studies used PAN as a basic radiological exam and a CT to confirm the presumptive diagnosis of BMC.

**Table 1:**
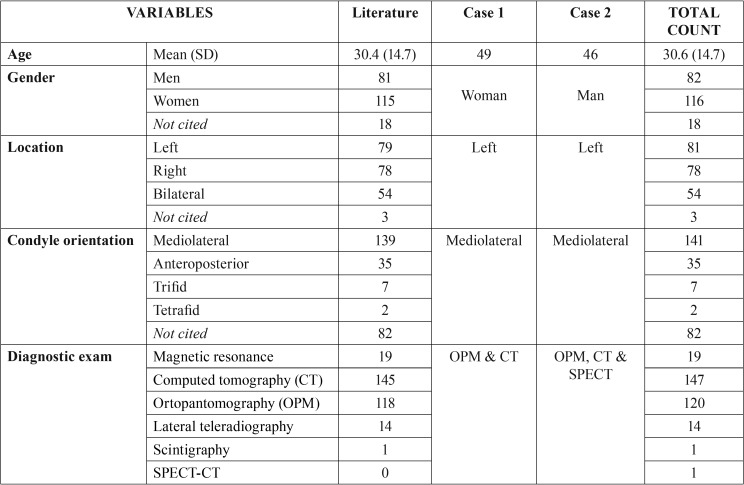
Demographic, clinicopathological and therapeutic characteristics of the cases included in the systematic review. DDR = reducing displaced disc; DDN = nonreducing displaced disc.

**Table 1 continue:**
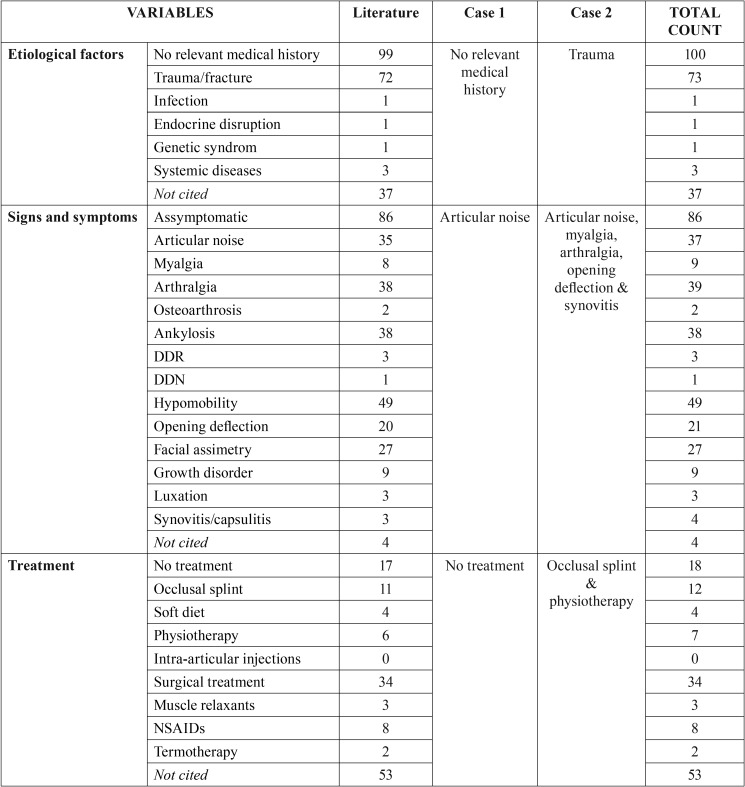
Demographic, clinicopathological and therapeutic characteristics of the cases included in the systematic review. DDR = reducing displaced disc; DDN = nonreducing displaced disc.

Although a great majority of patients were diagnosed of BMC as a casual finding, only the 40.6% were completely asymptomatic. From 71 asymptomatic cases with known etiology, 26 (36.6%) reported history of trauma. On the contrary, 63 from 126 symptomatic cases (50%) had history of trauma. However, from 69 cases with traumatic antecedents that reported signs and symptoms, 58 (84,1%) had symptoms and only 11 (15.9%) were asymptomatic.

This systematic review found 61 cases with history of trauma that reported condylar orientation. Among them, 51 (83.6%) were mediolateral, while 10 (16.4%) had an anteroposterior orientation. On the other hand, from cases with known etiology, 10 from 18 (55.5%) cases with anteroposterior orientation had trauma antecedents, while 51 from 114 (44.7%) with mediolateral condyle orientation reported history of trauma.

Among the symptomatic all patients, hypomobility (22.7%), arthralgia (18.1%), articular noise (17.2%) and ankylosis (17.6%) were the most frequent signs and symptoms. Specifically, 35 from 38 patients with ankylosis had history of trauma (92,2%).

The most common treatment options were active monitoring, manufacturing an occlusal splint and, at last, joint surgery in a 15.7% of all included cases as they were affected by ankylosis (78.6%), growth disorders (66.7%) or hypomobility (49%).

## Discussion

BMC is a rare anomaly that was first described by Hrdlicka in 1941 (79) and it is radiographycally characterized by a duplication of the mandibular condyle due to a groove of variable depth (13,14,29,45). Interestingly, trifid (13,27,55,65) and tetrafid (60) condyles have also been described.

Etiology and pathogenesis is not fully clarified (1,2,14,46,51,56,74). Although some consider BMC to be a developmental abnormality, traumatic origin is a common assumption in many studies (8,10,11,13,19,23,75,80). Artvinli and Kansu (27) published a clinical case of a 25 years old woman with bifid and trifid condyles with history of a traumatism that involved head and neck. Moreover, other authors have reported clinical cases of BMC with history of trauma in their childhood (13,65). In these cases, it is hypothesized the lesion could develop as a result of healing and remodeling after fractures of condylar regions (7,8,11-14,19,23,27,43,68,71).

Li *et al.* (23) reported 4 cases origined by fracture and classified its morphology by the severity of the trauma, location and relation with the lateral pterygoid muscle. This muscle affects the direction of the fractured condylar piece and it constitutes a relevant factor in creating a BMC (17,23,46,75). This theory is based on the fact that after a condylar neck fracture, an anteromedial displacement of the condyle is produced due to the lateral pterygoid muscle activity. Then, a new condylar head appears by metaplasia in a correct anatomic position, while the displaced condyle initiates a resorption process (32,71). Thus, from the two condyles, only the posterior would be functional (27,71).

Nevertheless, no signs of traumatic etiologic agents are reported in the great majority of published cases (1,9,10,12,13,22,26,39,45), including trifid (55,56,69) and tetrafid (60) condyles. This could suggest that some cases had a developmental abnormality. Hrdlicka (79) affirmed that the condyle divides as a result of blood supply blockage during the development. Blackwood (81) examined the condylar cartilage of 10 human skulls and found the presence of a well vascularized fibrous septum that disappeared two years after birth. This publication concluded that the presence of the septum along with a blood blockage could influence ossification, finally developing a BMC.

Some researchers suggested that mediolateral condyle orientation was associated to a non-traumatic etiology (fibrous septum), while anteroposterior position could be related to trauma (17,18). Nevertheless, mediolateral BMC after a condylar sagittal fracture have been described (8,11,15). Studies published by Balaji and Sampaio reported cases with mediolateral position and history of trauma (16,33). This study found that the 55.5% of condyles with an anteroposterior orientation and the 44.7% with a mediolateral position had history of trauma. However, mediolateral condylar orientation represented an 83.6% of all cases with trauma antecedents. According to Almasan *et al.* (22), the great majority of cases have a mediolateral orientation, independently from history of trauma or not. In fact, there are many factors that seem to be involved such as integrity and shape of the articular disk, the presence of a fibrous septum, the remodeling capability, muscular insertions, presence of fractures or displaced pieces and the articular capsule status.

This systematic review found that 40.6% of cases were asymptomatic, a similar value to the one published by Cho *et al.* (30) in a retrospective study. Asymptomatic cases are usually associated to a non-traumatic etiology and frequently detected during a routine examination (1). However, asymptomatic cases with history of trauma have also been described (23,33), and they represented a 36.6% in this systematic review. Even though, the present study found a great majority of cases with signs or symptoms (59,4%), being the most frequent: hypomobility (22,7%), arthralgia (18.1%), noises (17.2%) and ankyloses (17.6%). There are some symptomatic cases not associated to ankyloses or traumatisms presenting pain and articular noises (28,53), mouth opening decreasing (20), intermittent articular blockage and pain (22), or BMC diagnosed after a bilateral permanent luxation (24).

A 50% of symptomatic cases found in this study presented history of trauma, according to Hersek *et al.* (19), who presented a clinical case of a unilateral BMC with a click during the mouth opening in which the MRI did not found disk displacement. It is noteworthy that only a 15.9% of cases with trauma antecedents were asymptomatic. Szentpétery *et al.* (17) stated that in case of trauma, the apparition of symptoms related to a BMC were influenced by the lesion type (direct or indirect, and fracture position), extent of damage to articular structures, presence of swelling or hemarthrosis and age.

The relationship between BMC and ankylosis is rare (20,25), this study has found 38 (17.6%) cases. Interestingly, 35 from 38 cases (92.2%) with ankylosis had history of trauma. It is not possible to clarify if the condylar division was present before ankylosis or if this division could have influenced its development.

BMC cases are usually diagnosed by a panoramic radiography during a routine examination (1,12,14,19,26,31). However, in some cases it could hamper the detection of this pathology as it may have some distortion or magnification (22). In fact, some cases have been diagnosed posteriorly as PAN did show no signs of pathology (22,33). CT is considered as the test of choice (13,16,18,30,32). Not only provides more information about condylar morphology but also helps to perform differential diagnosis with tumors and primary osseous cysts, metastatic lesions, degenerative bony lesions and metabolic lesions that could also alter the morphology (28). Furthermore, it could be interesting to obtain functional information about mandibular condyles through other radiological tests such as Single Photon Emission Computed Tomography (SPECT), performed in Case 2 provided by the authors, to determine the potential of growth and the condyle remodeling to evaluate its prognosis and define the treatment plan. Interestingly, SPECT showed an asymmetry with more abstraction on the healthy condyle. This could be explained by an overload of such condyle due to orthopedic instability caused by the BMC.

No treatment is needed for asymptomatic BMC, although an active monitoring is recommended. Regarding symptomatic cases, distinct treatment could be applied depending on the type of symptoms. Non-steroidal anti-inflammatory drugs or analgesics, physiotherapy or occlusal splint are recommended as a conservative approach (13,22). Surgical treatment has only been described to restore function in BMC with ankyloses (20,40,65) or symptomatic cases resistant to conservative treatments (68).

The results of this study should be treated with caution because, according to SORT criteria, only level 3 studies were included. Furthermore, grey literature and manual search of journals were not performed. It is noteworthy that some cases in which no treatment was performed, were actually referred to patients that refused any kind of treatment.

## Conclusions

- Etiology of BMC could be congenital (developmental abnormality) or traumatic (healing after a condylar fracture).

- Computed tomography is the proof of choice to establish a correct diagnosis of BMC.

- Mediolateral condylar orientation is the most frequent position. No direct relationship between condylar orientation and etiology can be established although there is a tendency to observe more history of trauma in anteroposterior condylar positions.

- A 60% of cases are symptomatic, mainly reporting hypomobility and arthralgia. Half of them are related to trauma antecedents.

- The treatment options for symptomatic cases are usually conservative. Open surgery is only reserved for cases persistent to the initial treatment.
